# Pitfalls in the Diagnosis and Management of an Unusual Presentation of Clinically Amyopathic Dermatomyositis: A Case Report Written With the Assistance of ChatGPT

**DOI:** 10.7759/cureus.41879

**Published:** 2023-07-14

**Authors:** Jacqueline Jansz, Huynh W Tran, Nadera J Sweiss

**Affiliations:** 1 Internal Medicine, University of Illinois at Chicago, Chicago, USA; 2 Rheumatology, Wynn Medical Center, Rosemead, USA; 3 Rheumatology, University of Illinois at Chicago, Chicago, USA

**Keywords:** chatgpt, interstitial lung disease, ild, anti-mda5, amyopathic dermatomyositis, dermatomyositis, cadm, clinically amyopathic dermatomyositis

## Abstract

Clinically amyopathic dermatomyositis (CADM) is a rare form of dermatomyositis. Patients with this condition present with the typical skin findings of dermatomyositis but lack the characteristic muscle weakness associated with dermatomyositis. This case presentation highlights the unusual clinical manifestation of CADM in a 49-year-old Vietnamese female. The patient initially presented with persistent hyperpigmented plaques on her hands, which did not respond to the standard treatment for atopic dermatitis. The patient later developed respiratory failure and lung fibrosis in Vietnam. This case underscores the challenges in diagnosing and managing CADM, particularly in patients with atypical presentations, and emphasizes the difficulties in managing such cases of CADM in the community setting.

## Introduction

Clinically amyopathic dermatomyositis (CADM) is a rare subtype of dermatomyositis, an autoimmune disorder that commonly affects the skin and muscles. In CADM, patients have skin findings of dermatomyositis, but without muscle weakness for at least six months [1]. The condition is more frequent in Asian women [2].

Typical skin findings in CADM include a heliotrope rash, which is a purplish discoloration around the eyes, and Gottron papules, which are scaly, red patches on the knuckles and other bony prominences [3]. Other skin findings may include skin ulceration, poikiloderma (mottled discoloration), and photosensitivity [3].

Diagnosis of CADM can be challenging, as there are no specific laboratory tests for the condition. Diagnosis is typically based on a combination of clinical features, skin biopsy, and laboratory tests, such as muscle enzymes (creatine kinase), and autoantibodies, such as anti-melanoma differentiation-associated gene 5 (anti-MDA5) antibody [4].

CADM is associated with interstitial lung disease (ILD), which can cause breathing difficulties and requires close monitoring. Thus, the importance of monitoring in CADM is critical, as early detection and treatment of complications may improve outcomes. Regular monitoring may include pulmonary function tests, imaging studies such as chest computed tomography (CT) scans, and regular laboratory tests to monitor muscle enzymes and autoantibodies [5,6].

## Case presentation

A 49-year-old Vietnamese female presented to the clinic with a history of rash on her face and hands, extending from her fingertips to her wrists for the past month. She did not have any weight loss, mouth ulcers, Raynaud's phenomenon, arthralgia, weakness, photosensitivity, alopecia, fever, or changes in bowel habits. Her medical history was significant for hypertension. She did not have any significant family history, smoking, or alcohol history.

**Figure 1:**
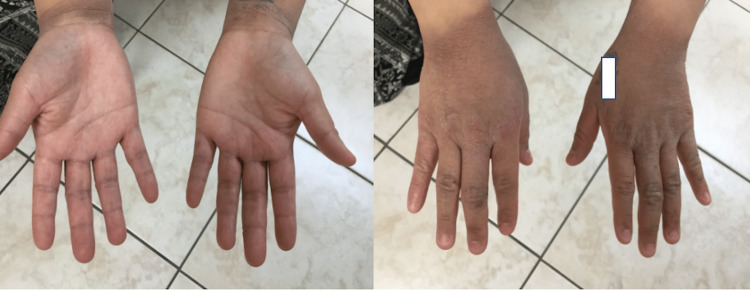
Hyperpigmented plaques in a glove-like distribution

During the initial visit, the patient’s vitals were stable with a blood pressure of 147/98. On the physical exam, she had hyperpigmented patches, extending from her fingers all the way to her wrists in a glove-like distribution bilaterally (Figure 1). Muscle strength was intact in the upper and lower extremities bilaterally.

She was initially diagnosed with atopic dermatitis and started on hydrocortisone 2.5% ointment without improvement. She was then tried on clobetasol propionate 0.05% ointment, again without improvement. The decision was made to start systemic treatment for atopic dermatitis with oral cyclosporine. Her rash started to decrease with cyclosporine 100 mg daily. In subsequent visits, cyclosporine was increased to 200 mg daily with more improvement but did not completely resolve her rash.

Several months later, the patient came back to the clinic with shortness of breath, fatigue on exertion, muscle weakness, and a new lump within the left breast. Of note, she had no history of previous mammograms. The rash had improved but still had not resolved completely. On exam, the patient had clear lung sounds, decreased hyperpigmented plaques on the proximal palmar hand, and a 4 cm non-tender nodule at the four o'clock position on the left breast. She had ⅘ strength in the proximal muscles and ⅘ strength in the quadriceps. She was able to stand but needed assistance. She had been switched to dupilumab 300 mg subcutaneous injection every two weeks by a provider at another clinic. Imaging was also ordered during this visit, including a chest CT without contrast, a referral to cardiology for echocardiography, and an order for a mammogram. Unfortunately, the patient did not have the imaging done.

Due to the lack of response to standard atopic dermatitis treatment, additional labs were ordered. Her labs are demonstrated below (Table 1).

**Table 1:** Patient's lab values

Test result	Patient	Reference range	Unit
Erythrocyte sedimentation rate (ESR)	55	0-20	mm/hr
C-reactive protein (CRP)	Negative	Negative	-
Antinuclear antibodies (ANA)	Negative	Negative	-
Creatine phosphokinase (CPK)	249	24-170	U/L
Vitamin D	12.8	>30	ng/mL
Lactate dehydrogenase (LDH)	493	94-250	U/L
Aldolase	12.2	<8.1	U/L
Jo-1 antibodies	11	<11	SI
PL-7 antibodies	11	<11	SI
PL-12 antibodies	11	<11	SI
EJ antibodies	11	<11	SI
OJ antibodies	11	<11	SI

Unfortunately, the patient left the United States and went to Vietnam prior to the results of her antisynthetase panel. The patient was never tested for anti-MDA5 antibodies.

A few weeks later, the patient ended up in the intensive care unit in Vietnam with progressive respiratory failure and lung fibrosis.

## Discussion

CADM is a subtype of dermatomyositis that predominantly affects the skin without muscle weakness or myositis. The prevalence of CADM is estimated to be around 10% of all dermatomyositis cases and is higher in Asians [2,7].

Early diagnosis of CADM is crucial because complications can include rapidly progressive ILD or acute respiratory distress syndrome (ARDS), which can be life-threatening. Patients with CADM who test positive for anti-MDA5 antibodies are at a higher risk of developing ILD and ARDS [8]. Although this patient did not complete the anti-MDA 5 antibody testing often associated with ILD in patients with CADM. It is important to note that not all patients with CADM exhibit these antibodies [9]. This highlights the need for additional biomarkers and diagnostic approaches to accurately identify and confirm CADM cases in patients without detectable anti-MDA5 antibodies.

The management of CADM is challenging, and a multidisciplinary team approach involving rheumatologists, pulmonologists, dermatologists, and other specialists is essential. Treatment typically involves the use of immunosuppressive agents, such as corticosteroids, methotrexate, or mycophenolate mofetil [10]. However, in the case presented, the patient expressed that she was uncomfortable receiving care at an academic center and preferred a clinic where the providers spoke her native language, i.e., Vietnamese. This preference hindered her access to a multidisciplinary team that could have optimized her care.

Additionally, the patient lacked insurance coverage, which heightened her concerns about medical tests and expenses. The high cost of antibody tests, imaging studies, and biopsies posed barriers to timely diagnosis and impeded her access to necessary healthcare resources. Patients in similar circumstances could benefit from compassionate care resources available at academic centers, which assist individuals with limited or no insurance. Moreover, even with an accurate diagnosis, the treatment of CADM can be expensive.

Patients with CADM are at an increased risk for malignancies, particularly breast, ovarian, lung, and hematologic cancers [11]. The presence of a new breast lump, in this case, raised concerns about potential malignancy; however, due to limited follow-up and imaging resources, a proper evaluation for breast pathology was not conducted.

Further research is necessary to better understand the pathogenesis of CADM and to develop more diagnostic and treatment strategies that are cost-effective. In particular, identifying additional biomarkers that may be associated with ILD or ARDS in CADM could improve the accuracy of diagnosis and guide treatment decisions. Additionally, research into the long-term outcomes of CADM is essential to determine the optimal management strategies for this rare condition in different demographic populations.

**Figure 2:**
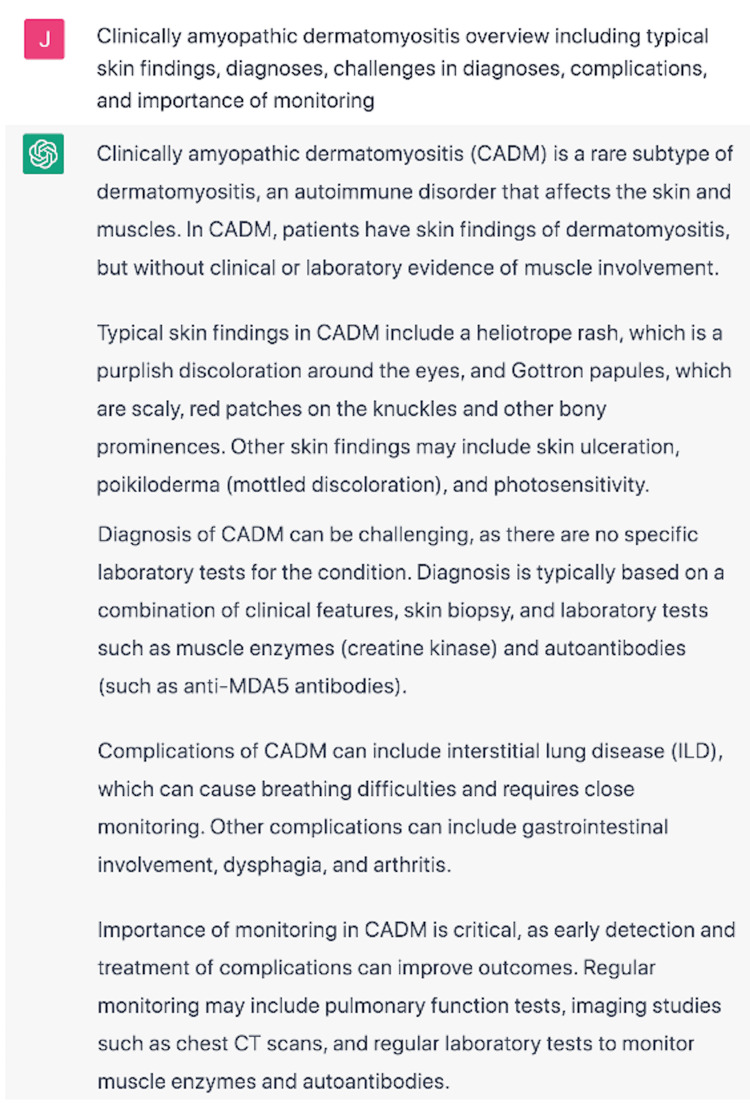
Query used for ChatGPT to provide a summary

**Figure 3:**
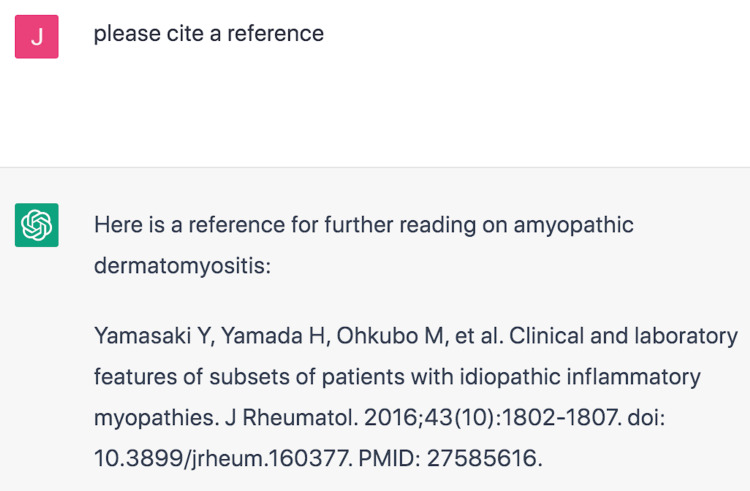
Fake citation generated by ChatGPT

ChatGPT is an artificial intelligence chatbot produced by OpenAI (San Francisco, CA) to simulate conversation with humans. Among the things that ChatGPT claims to do include explaining topics and summarizing information. However, can it be used to accurately summarize medical literature for a case report? In my experience writing this case report, ChatGPT was able to summarize topics (Figure 2), but at times generated false literature (Figure 3). Although ChatGPT may be useful, it is still in its early stages and needs to be used carefully with an expert review of the information it is providing. Looking to the future, ChatGPT will need to be able to more accurately source and cite literature to prove useful as an aide in summarizing medical literature for a case report.

## Conclusions

In conclusion, CADM presents diagnostic and management challenges, particularly in patients with atypical presentations and limited access to specialized care. Issues such as language barriers, financial constraints, and the unavailability of specific tests can impede timely diagnosis and appropriate treatment. Efforts should be made to improve accessibility to diagnostic resources, promote culturally sensitive care, expand compassionate care initiatives, and explore cost-effective approaches for the diagnosis and management of CADM. Further research is crucial to advance our understanding of CADM and develop strategies that optimize patient outcomes, particularly in cases where anti-MDA5 antibodies are not detectable or not available.
